# Demographics and Characteristics of Patients Admitted With Acute Coronary Syndrome to the Coronary Care Unit at King Abdulaziz University

**DOI:** 10.7759/cureus.26113

**Published:** 2022-06-20

**Authors:** Siba Z Takieddin, Naif M Alghamdi, Mansour S Mahrous, Bader M Alamri, Qusai A Bafakeeh, Mohammed A Zahrani

**Affiliations:** 1 Medicine and Surgery, King Abdulaziz University Hospital, Jeddah, SAU; 2 Cardiology, King Abdulaziz University Hospital, Jeddah, SAU

**Keywords:** percutaneous coronary intervention, retrospective studies, non-st elevation myocardial infraction, unstable angina, st-elevation myocardial infarction (stemi), myocardial infarction, saudi arabia, coronary care unit, cardiovascular diseases, acute coronary syndrome

## Abstract

Background

Over the previous decade, the incidence of cardiovascular diseases (CVDs) has risen in the Middle East and will increase mortality to 23 million individuals in Saudi Arabia by 2030, according to the Saudi Ministry of Health. CVDs, including acute coronary syndrome (ACS), are the most common cause of mortality globally. This study aimed to analyze the demographic and clinical characteristics of patients with ACS admitted to the coronary care unit (CCU) in a tertiary hospital in Jeddah, Saudi Arabia. To the best of our knowledge, a lack of research in this region has been undertaken.

Methods

This retrospective records review study was conducted in a tertiary center in Jeddah, Saudi Arabia. All patients admitted to our CCU in 2017 with a final diagnosis of ACS were retrospectively enrolled. Demographic details, coronary risk factors, investigation and procedures, management, and clinical outcomes are all part of the data.

Results

Of the 615 patients included in the study, 491 (79.84%) were males, 226 (36.75%) were 55-64 years old, and 161 (26.18%) were 45-54 years old. Males had a higher rate of ST-segment elevation myocardial infarction (STEMI) (214, 43.58%), while females had a higher rate of non-ST-segment elevation myocardial infarction (NSTEMI) and unstable angina (UA) (45.96% and 37.90%, respectively). Diabetes (62.60%), dyslipidemia (62.44%), and hypertension (61.46%) were the most prevalent risk factors. Angiography and percutaneous coronary intervention (PCI) were performed in 77.72% and 61.95% of patients, respectively. Coronary artery bypass graft was only performed in 4.39% of patients. PCI was performed more frequently in patients with STEMI than in those with NSTEMI/UA (P < 0.001). A large majority of patients (99.5%) recovered and were discharged. Of the 161 (26.18%) patients who attended a follow-up visit, only 45 (33.08%) met the therapeutic objective of 1.8 mmol/L (70 mg/dl) of low-density lipoprotein cholesterol. There were 100 (16.26%) patients readmitted to the CCU, and most of these were readmitted within a year after initial admission. Readmissions were more common in females and patients diagnosed with NSTEMI/UA during initial admission (15.47% and 19.35%, respectively).

Conclusion

This study revealed that our most common demographics were males between 45 and 64 years, which is a decade younger than the global average. STEMI was the most common presentation. The most common modifiable cardiovascular risk factors were hypertension, diabetes, and dyslipidemia. The most common adverse event was reinfarction, which was closely linked to hypertension and diabetes. In this study, the recovery rate was higher than in studies from other countries; however, the majority of patients did not achieve the goal of cholesterol levels at follow-up. Our population's younger age at presentation necessitates greater attention and more stringent preventive strategies, such as lifestyle changes and evidence-based treatments for CVD risk factors, to reduce the incidence and burden of ACS on CCUs.

## Introduction

Cardiovascular diseases (CVDs) are the leading cause of death worldwide, claiming an estimated 17.9 million lives annually, according to the World Health Organization [1]. CVD refers to a set of disorders that affect the heart and its blood vessels and can be further categorized into coronary artery disease (CAD) and acute coronary syndrome (ACS) [2]. CAD is defined by atherosclerosis in the coronary arteries, wherein atherosclerotic plaque builds up inside the coronary arteries, restricting blood circulation, and hence, delivery of oxygen to the heart; while CAD can be asymptomatic, ACS is characterized by signs and symptoms of sudden myocardial ischemia caused by CAD [3,4]. ACS is classified as unstable angina (UA), ST-segment elevation myocardial infarction (STEMI), or non-ST-segment elevation myocardial infarction (NSTEMI) [2,5].

The incidence of CVD has increased in the Middle East over the last decade, with numerous studies indicating that CVDs are prevalent in the region [6]. According to the Ministry of Health in Saudi Arabia, CVDs will claim the lives of approximately 23 million people by 2030 [7]. The population of Saudi Arabia and other neighboring Gulf countries mainly carry preventable risk factors due to rapid socio-economic growth, resulting in a massive shift in lifestyle, such as increased intake of low-quality cholesterol-laden meals and adoption of a sedentary lifestyle, which has led to an increase in CVD rates [6,8,9].

The primary reason for coronary care unit (CCU) admission was ACS, as established in a previous single-center study conducted in Saudi Arabia [10]. Since the 1960s, CCUs have been linked to decreased mortality in patients with ACS [11-19]. This is considered associated with more frequent prescriptions of evidence-based medicines and more rigorous monitoring, rapid detection, and treatment of life-threatening arrhythmias [11,17]. However, caring for critically ill patients is unquestionably one of the most challenging and time-consuming elements of intensive care medicine, and the CCU incurs high costs for both health institutions and the medical staff [10,20]. Saudi Arabia has different regions with varying patient demographics, clinical characteristics, management, and quality of care. Exploring these elements may help implement more effective approaches to the prevention and management of ACS and potentially improve healthcare systems, thus minimizing the burden on the CCU. To the best of our knowledge, no studies of this nature have been conducted in Saudi Arabia's western region. Therefore, this study aimed to examine and analyze the demographic and clinical features, management, and outcomes of ACS patients admitted to the CCU at King Abdulaziz University Hospital (KAUH) in Jeddah, Saudi Arabia.

## Materials and methods

Study setting and participants

This study was a retrospective review of medical records performed in June 2021 at KAUH, a tertiary care center in Jeddah, Saudi Arabia. Between January and December 2017, 673 patients were admitted to our CCU, which includes 10 well-equipped beds for patients with acute cardiac conditions. Of those, we enrolled 615 patients who met the ACS criteria. The diagnosis of ACS was established based on the patient’s clinical presentation (ischemic signs or symptoms compatible with ACS) associated with any of the following: changes in the electrocardiogram (ECG) suggestive of ACS, increase in biochemical markers of cardiac necrosis (creatine phosphokinase, troponin, and creatine kinase-MB), or confirmed CAD. Patients with congenital cardiac abnormalities, incomplete data records, or ACS due to a non-cardiovascular etiology (e.g. trauma or surgery) were excluded.

Evaluated indicators

The hospital records of selected patients were reviewed for baseline demographic characteristics, such as age, sex, nationality, and medical history, including significant coronary risk factors, such as smoking, presence of diabetes mellitus (DM), dyslipidemia, hypertension, obesity, previous ACS event or cerebrovascular accident, congestive heart failure, percutaneous coronary intervention (PCI), or coronary artery bypass graft (CABG). To ensure data consistency, standard definitions were used as follows: (1) smoking status: current smokers (individuals who smoked every day or some days at presentation), non-smokers (people who had never smoked more than 100 cigarettes in their lives), and ex-smokers (individuals who had quit smoking 30 days prior to admission); (2) hypertension: self-reporting of previous hypertension diagnosis or use of anti-hypertensive medications; (3) DM: self-reporting of previous DM diagnosis or use of anti-diabetic medications; (4) dyslipidemia: total cholesterol (TC) > 5.18 mmol/L, low-density lipoprotein cholesterol (LDL-C) > 2.59 mmol/L, or non-high-density lipoprotein cholesterol (non-HDL-C) > 3.37 mmol/L [21]. Obesity is defined as a body mass index (BMI) ≥ 30 kg/m^2^ determined using the Quetelet index formula (mass in kg/height in m^2^).

Additionally, a comprehensive analysis of lipid profiles and lipid-lowering agents used during admission and follow-up was performed. The therapeutic target value of LDL-C was measured according to the European Society of Cardiology/European Atherosclerosis Society (ESC/EAS) guidelines [22]. Details of hospitalization procedures were recorded, and echocardiographic findings were obtained from final reports confirmed by a cardiologist. The American College of Cardiology's criteria were used to classify the data on left ventricular ejection fraction (LVEF) [23]. Moreover, in-hospital outcomes, including major adverse cardiovascular events, such as death, resuscitated cardiac arrest, reinfarction, stroke, and major bleeding, were also noted. Finally, readmissions for a new acute coronary event (STEMI, NSTEMI, or UA) during 2017 and 2018, including lipid profiles and medications administered, were recorded and analyzed.

Ethical approval

The study protocol strictly followed standard clinical guidelines and was approved by the Research Ethics Committee (REC) of KAUH (reference number: 299-20). Due to the deidentified nature of the databases, written informed consent was not required; however, each patient's medical record number was identified and patient confidentiality was preserved.

Statistical analysis

Data were extracted from the hospital’s patient database (Phoenix) and entered into Microsoft Excel 2016 (Microsoft Corporation, Redmond, WA). Quantitative variables are presented as mean ± standard deviation, whereas categorical variables are presented as percentages. The Student’s t-test and the chi-square test were used to compare the continuous and dichotomous variables groups, respectively. Statistical significance was set at P < 0.05, and IBM SPSS Statistics for Windows (version 21; IBM Corp., Armonk, NY) was used for all statistical analyses.

## Results

Demographic and coronary risk factors profile

In total, 615 patients who had been diagnosed with ACS were enrolled in this retrospective chart review study, and their data were analyzed. Most patients in our study sample were males (79.84%), non-Saudis (76.58%), and between 55 and 64 years old (36.75%) and 45 and 54 years old (26.18%). The most prevalent modifiable cardiac risk factors were DM (62.60%), dyslipidemia (62.44%), and hypertension (61.46%). Subsequently, the patients were separated into three groups as follows: STEMI, NSTEMI, and UA. STEMI was the most common presentation at first admission (38%), followed by NSTEMI (35%). The baseline demographics and coronary risk factors of these patients are shown in Table 1.

**Table 1:** Baseline demographic and clinical characteristics of patients with acute coronary syndrome * Chi-square; ^†^ One-way ANOVA test. ACS = acute coronary syndrome; BMI = body mass index; bpm = beats per minute; CABG = coronary artery bypass graft; CVA = cerebrovascular accident; DBP = diastolic blood pressure; NSTEMI = non-ST-segment elevation myocardial infarction; PCI = percutaneous coronary intervention; SBP = systolic blood pressure; SD = standard deviation; STEMI = ST-segment elevation myocardial infarction; UA = unstable angina.

Characteristics	Overall (n = 615)	STEMI (n = 234)	NSTEMI (n = 215)	UA (n = 166)	P-value	
						Gender, n (%)						
Males	491 (79.84)	214 (91.45)	158 (73.49)	119 (71.69)	<0.001^*^	
Females	124 (20.16)	20 (8.55)	57 (26.51)	47 (28.31)		
Age (years), mean ±SD						
Total	57.60 ± 10.99	55.91 ± 10.83	59.98 ± 11.33	56.91 ± 10.30	<0.001^†^	
Males	59.70 ± 10.59	55.79 ± 10.84	58.30 ± 10.47	56.21 ± 10.12	0.066^†^	
Females	64.16 ± 11.87	57.10 ± 10.90	64.63 ± 12.37	58.68 ± 10.64	0.009^†^	
Age group, n (%)						
<45	75 (12.20)	38 (16.24)	16 (7.44)	21 (12.65)	0.001*	
45-54	161 (26.18)	70 (29.91)	52 (24.19)	39 (23.49)		
55-64	226 (36.75)	73 (31.20)	84 (39.07)	69 (41.57)		
65-74	111 (18.05)	43 (18.38)	36 (16.74)	32 (19.28)		
75-84	34 (5.53)	8 (3.41)	22 (10.23)	4 (2.41)		
≥85	8 (1.30)	2 (0.85)	5 (2.33)	1 (0.60)		
Nationality, n (%)						
Saudi	144 (23.41)	45 (19.23)	47 (21.86)	52 (31.33)	0.015^*^	
Non-Saudi	471 (76.59)	189 (80.77)	168 (78.14)	114 (68.67)		
BMI, n (%)						
Underweight	8 (1.30)	2 (0.85)	3 (1.40)	3 (1.81)	<0.001*	
Normal	220 (35.83)	104 (44.44)	73 (34.11)	43 (25.90)		
Pre-obesity	228 (37.13)	89 (38.03)	79 (36.92)	60 (36.14)		
Obesity class I	116 (18.89)	33 (14.10)	44 (20.56)	39 (23.49)		
Obesity class II	33 (5.37)	3 (1.28)	14 (6.54)	16 (9.64)		
Obesity class III	9 (1.47)	3 (1.28)	1 (0.47)	5 (3.01)		
Vital signs at the time of arrival/admission, mean ±SD						
SBP (mmHg)	143.08 ± 28.57	139.15 ± 30.21	145.84 ± 28.14	145.08 ± 26.17	0.029^†^	
DBP (mmHg)	80.63 ± 17.04	81.82 ± 17.19	80.73 ± 18.69	78.82 ± 14.28	0.234^†^	
Heart rate (bpm)	85.36 ± 21.03	85.43 ± 20.28	88.05 ± 22.79	81.68 ± 19.18	0.015^†^	
Past medical history, n (%)						
Diabetes	385 (62.60)	136 (58.12)	142 (66.05)	107 (64.46)	0.188^*^	
Hypertension	378 (61.46)	118 (50.43)	143 (66.51)	117 (70.48)	<0.001^*^	
Dyslipidemia	384 (62.44)	168 (71.79)	129 (60.00)	87 (52.41)	<0.001^*^	
Obesity	159 (25.85)	39 (16.67)	60 (27.91)	60 (36.14)	<0.001^*^	
Previous ACS episode	166 (27.99)	34 (14.53)	63 (29.30)	69 (41.57)	<0.001^*^	
Previous heart failure	8 (1.30)	1 (0.43)	7 (3.26)	0 (0.00)	0.007^*^	
Previous CVA	8 (1.30)	3 (1.28)	4 (1.86)	1 (0.60)	0.545^*^	
Previous PCI	109 (17.72)	24 (10.26)	39 (18.14)	46 (27.71)	<0.001^*^	
Previous CABG	30 (4.88)	4 (1.71)	17 (7.91)	9 (5.42)	0.009^*^	
Smoking status, n (%)						
Ex-smoker	96 (15.61)	29 (12.39)	36 (16.74)	31 (18.67)	0.199^*^	
Current smoker	173 (28.13)	85 (36.32)	52 (24.19)	36 (21.69)	0.002^*^	
Non-smoker	346 (56.26)	120 (51.28)	127 (59.07)	99 (59.64)	0.148^*^	

Males mostly presented with STEMI (43.68%), compared with females who mostly presented with NSTEMI and UA (45.96% and 37.90%, respectively, P < 0.001). Incidence of obesity was significantly higher among the NSTEMI and UA groups (37.74% and 37.74%, respectively) than among the STEMI group (24.53%, P ≤ 0.001), with a statistically significant sex-specific difference (29.56% of females compared to 70.44% of males, P = 0.001). Remarkably, the occurrence of previous ACS episodes was higher among the NSTEMI and UA groups (P < 0.001), with a noted sex disparity (37.1% of females compared to 24.4% of males, P = 0.017). The incidence of hypertension was noticeably higher in the NSTEMI group (37.83%, P < 0.001), whereas smoking and dyslipidemia were notably more prevalent among patients with STEMI (49.13% and 43.75%, respectively, P = 0.002 and P ≤ 0.001). History of previous heart failure (P = 0.007) or CABG (P = 0.009) was more frequent in the NSTEMI group.

Lipid profile

Analysis of the lipid profile showed that the mean values of TC, LDL-C, triglycerides (TG), and high-density lipoprotein (HDL) were 4.59 ± 1.21, 2.99 ± 1.02, 1.93 ± 1.19, and 1.04 ± 0.35 mmol/L, respectively. Moreover, the mean lipid profile values were found to differ according to sex in our study. TC (P = 0.039), low-density lipoprotein (LDL) (P = 0.002), and TG (P = 0.001) were remarkably higher in males than in females. However, mean HDL levels were significantly higher in females than in males (P < 0.001) (Table 2).

**Table 2:** Gender differences between lipid parameters at hospital admission * Independent t-test. HDL-C = high-density lipoprotein cholesterol; LDL-C = low-density lipoprotein cholesterol; SD = standard deviation.

Variables	Males (mean ± SD)	Females (mean ± SD)	P-value
LDL-C, mmol/L	3.05 ± 1.03	2.72 ± 0.95	0.002^*^
Total cholesterol, mmol/L	4.64 ± 1.21	4.39 ± 1.18	0.039^*^
Triglyceride, mmol/L	1.99 ± 1.27	1.70 ± 0.77	0.001^*^
HDL-C, mmol/L	1.01 ± 0.34	1.15 ± 0.35	<0.001^*^

Of the 615 patients, 161 (26.18%) returned to the hospital for follow-up visits, and their lipid profile values were analyzed. The mean LDL (P < 0.001), TC (P < 0.001), and TG (P = 0.002) were significantly lower at follow-up than at hospital admission, with a consistent pattern observed for both sexes (Table 3).

**Table 3:** Comparison of lipid parameters between hospital admission and follow-up * Paired t-test. HDL-C = high-density lipoprotein cholesterol; LDL-C = low-density lipoprotein cholesterol; SD = standard deviation.

Variables	Hospital admission (mean ± SD)	Follow-up (mean ± SD)	P-value
LDL-C, mmol/L	2.97 ± 0.94	2.36 ± 1.02	<0.001^*^
Total cholesterol, mmol/L	4.63 ± 1.20	3.82 ± 1.30	<0.001^*^
Triglyceride, mmol/L	2.07 ± 1.56	1.72 ± 0.99	0.002^*^
HDL-C, mmol/L	1.07 ± 0.31	1.05 ± 0.28	0.507^*^

The therapeutic target of LDL-C < 1.8 mmol/L (<70 mg/dl), according to the ESC/EAS guidelines [22], was achieved in 45 (33.08%) patients. The NSTEMI group had the most effective treatment, with 20 (40%) patients meeting the therapeutic aim. In the UA group, the target was achieved in 12 (35.29%) patients, followed by only 13 (25%) patients with STEMI.

Treatment modalities and procedures performed

**Figure 1:**
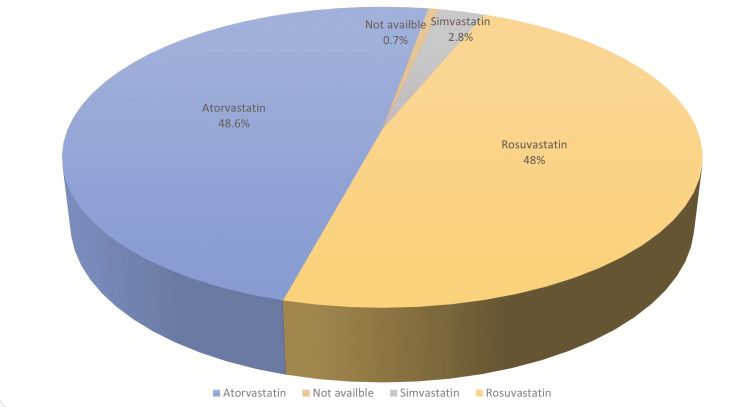
The rate of statin use during hospitalization includes dosages from 10 to 80 mg/day

In terms of pharmacological therapy administered during hospitalization, all patients received lipid-lowering agents. Overall, 594 (96.59%) patients were managed with atorvastatin or rosuvastatin (Figure 1).

**Figure 2:**
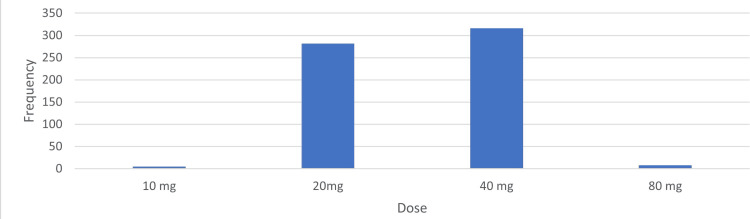
Dosages of lipid-lowering agents used at first admission

Furthermore, the largest proportion of doses administered was 40 mg/dl (51.72%), followed by 20 mg/dl (46.15%) (Figure 2).

Among females, rosuvastatin treatment (46.77%) was predominantly administered compared to males, who were mostly treated with atorvastatin (49.49%, P = 0.010). During follow-up, patients treated with 40 mg/day of atorvastatin had significantly lower average levels of LDL (P < 0.001), TC (P < 0.001), and TG (P = 0.023) than those treated with 20 mg/day of atorvastatin (Table 4).

**Table 4:** The effect of atorvastatin and rosuvastatin on lipid profile parameters * Paired t-test. HDL = high-density lipoprotein; LDL = low-density lipoprotein; SD = standard deviation; TC = total cholesterol.

Variables	Lipid profile values at admission (mean ± SD)	Lipid profile values at follow-up (mean ± SD)	P-value
Atorvastatin (20 mg/day)			
LDL, mmol/L	2.13 ± 0.96	2.17 ± 0.74	0.920*
TC, mmol/L	3.80 ± 1.13	3.63 ± 0.82	0.693*
Triglyceride, mmol/L	1.76 ± 0.80	1.57 ± 0.65	0.676*
HDL, mmol/L	0.98 ± 0.20	1.12 ± 0.36	0.477*
Atorvastatin (40 mg/day)			
LDL, mmol/L	2.90 ± 0.81	2.22 ± 0.87	<0.001*
TC, mmol/L	4.58 ± 1.19	3.69 ± 1.19	<0.001*
Triglyceride, mmol/L	2.19±1.82	1.67 ± 0.93	0.023*
HDL, mmol/L	1.03 ± 0.30	0.99 ± 0.24	0.466*
Rosuvastatin (20 mg/day)			
LDL, mmol/L	3.10 ± 1.05	2.46 ± 1.12	<0.001*
TC, mmol/L	4.71 ± 1.26	3.86 ± 1.39	<0.001*
Triglyceride, mmol/L	2.05 ± 1.48	1.73 ± 0.93	0.015*
HDL, mmol/L	1.07 ± 0.31	1.07 ± 0.31	0.979*
Rosuvastatin (40 mg/day)			
LDL, mmol/L	2.83 ± 0.63	2.56 ± 1.30	0.607*
TC, mmol/L	4.72 ± 0.67	4.15 ± 1.30	0.401*
Triglyceride, mmol/L	1.22 ± 0.50	1.59 ± 0.68	0.348*
HDL, mmol/L	1.37 ± 0.44	1.16 ± 0.31	0.133*

In contrast, the mean lipid levels in patients treated with 20 mg/day of rosuvastatin at follow-up were significantly lower than those in patients treated with 40 mg/day of rosuvastatin: LDL (P < 0.001), TC (P < 0.001), and TG (P = 0.015). There was no significant difference in HDL levels throughout follow-up compared to admission values with either medication (P > 0.05).

**Figure 3:**
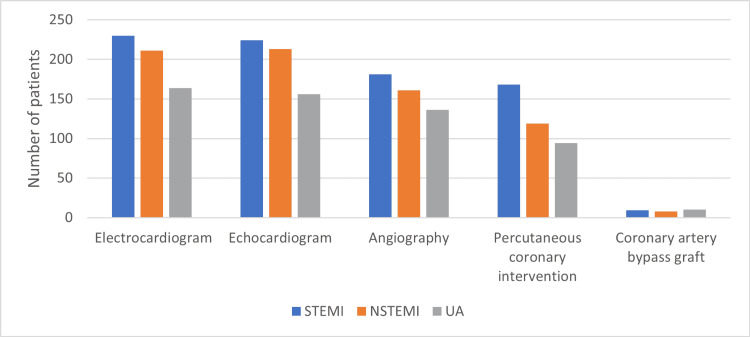
In-hospital management STEMI = ST-segment elevation myocardial infarction; NSTEMI = non-ST-segment elevation myocardial infarction; UA = unstable angina.

Regarding procedures performed during hospitalization, electrocardiography followed by echocardiography were the most common procedures performed during hospital stay (98.37% and 96.42%, respectively). For invasive procedures, angiography and PCI were performed in 77.72% and 61.95% of the patients, respectively. In contrast, CABG was performed in only 4.39% of patients. In terms of ACS type, PCI was used more frequently in patients with STEMI than in those with NSTEMI/UA (P < 0.001) (Figure 3).

Males underwent catheterization more often than females (46.84% of males compared to 34.67% of females, P = 0.020).

Echocardiography findings

For 573 out of the 615 study records, information on LVEF evaluated by left ventriculography or echocardiogram during the index hospitalization was available. The overall midpoint LVEF of the study was 47 ± 12.8%. It was divided by the dysfunctional severity shown in Table 5.

**Table 5:** Assessment of left ventricular ejection fraction during hospitalization * Chi-square; ^†^ one-way ANOVA test. LVEF = left ventricular ejection fraction; SD = standard deviation; STEMI = ST-segment elevation myocardial infarction; NSTEMI = non-ST-segment elevation myocardial infarction; UA = unstable angina.

Variables	Overall	STEMI	NSTEMI	UA	P-value
LVEF, mean ± SD	47.01 ± 12.76	43.87 ± 10.89	46.84 ± 13.10	51.73 ± 13.42	<0.001^†^
Hyperdynamic (>70%), n (%)	18 (3.14)	2 (0.92)	4 (1.96)	12 (7.95)	
Normal (50-70%), n (%)	247 (43.11)	60 (27.52)	103 (50.49)	84 (55.63)	
Left ventricular dysfunction, n (%)					<0.001*
Mild (40-49%)	146 (25.48)	84 (38.53)	38 (18.63)	24 (15.89)	
Moderate (30-39%)	115 (20.07)	59 (27.06)	33 (16.18)	23 (15.23)	
Severe (<30%)	47 (8.20)	13 (5.96)	26 (12.74)	8 (5.30)	

**Figure 4:**
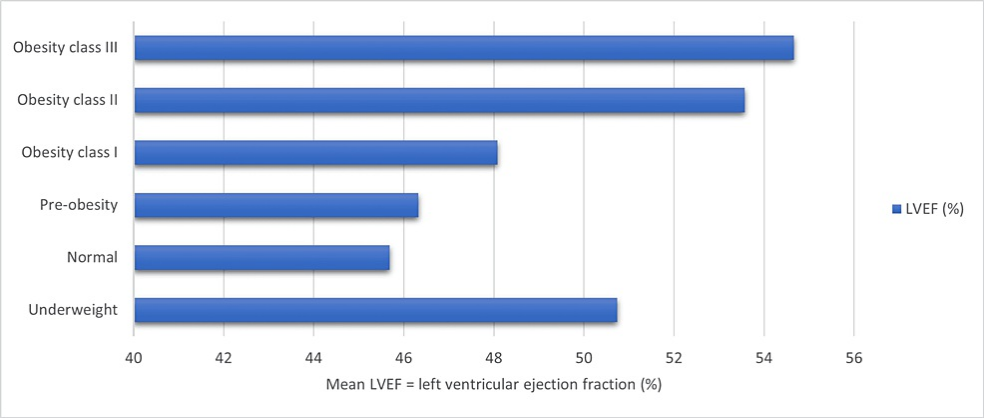
A comparative analysis of left ventricular ejection fraction (%) across different weight classes

Hyperdynamic LVEF was mostly noted in the UA group. In contrast, the incidence of mild and moderate left ventricular (LV) dysfunction was higher in the STEMI group, while that of severe dysfunction was higher in the NSTEMI group (P < 0.001). Females who presented with abnormal LVEF mostly had moderate LV dysfunction (40%), whereas males commonly had mild LV dysfunction (48.34%, P < 0.001). The high-risk (class III) obesity group showed a significantly higher mean ejection fraction than the normal weight group (P = 0.038) (Figure 4).

Outcomes

**Figure 5:**
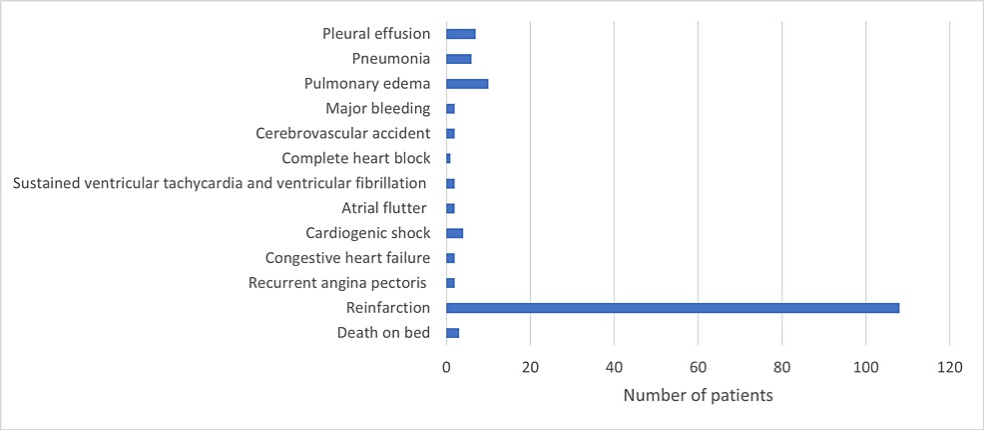
In-hospital outcomes

Most of the patients (99.5%) recovered and were discharged from the CCU without adverse hospital events. The most common adverse event was reinfarction in 108 patients (17.6%) (Figure 5).

The rate of reinfarction was significantly higher among patients on lipid-lowering medications before their first hospital admission than among those who were not on medication (22.97% and 10.61%, respectively, P = 0.012).

**Figure 6:**
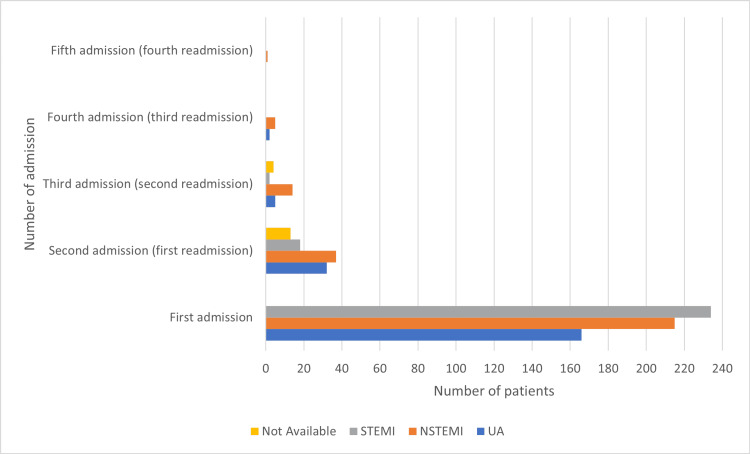
Number of coronary care unit readmissions for patients admitted in 2017 with the acute coronary syndrome and followed up until 2018 STEMI = ST-segment elevation myocardial infarction; NSTEMI = non-ST-segment elevation myocardial infarction; UA = unstable angina.

A total of 100 (16.26%) patients were readmitted to the CCU, and most of the readmissions were within 12 months, usually one to two months, after the first admission. The readmitted patients were mainly diabetic and hypertensive and between 55 and 64 years old. The readmission rate was higher among patients diagnosed with NSTEMI/UA during the first admission (Figure 6).

The readmission percentage was higher among females than among males (19.35% and 15.47%, respectively). Patients on lipid-lowering medications before hospital admission had a higher percentage of readmission than medically free patients (20.27% and 9.49%, respectively, P = 0.014). NSTEMI was the most common presentation among all readmissions.

Second admission

At the time of the second ACS hospital admission, the mean LDL-C (P < 0.001) and TC (P < 0.001) readings were considerably lower than those at the time of the first admission; in contrast, TG levels were higher during the second admission. Compared to the previous admission, there was a minor increase in TG, compared with HDL levels that slightly decreased (Table 6).

**Table 6:** Lipid profile during second hospital admission HDL = high-density lipoprotein; LDL = low-density lipoprotein; SD = standard deviation.

Variables	Mean ± SD	P-value
LDL, mmol/L	2.21 ± 0.95	<0.001
Total cholesterol, mmol/L	3.74 ± 1.17	<0.001
Triglyceride, mmol/L	2.06 ± 1.35	0.496
HDL, mmol/L	0.98 ± 0.26	0.092

The most commonly prescribed lipid-lowering medication during the second admission was atorvastatin (61.22%), followed by rosuvastatin (37.76%) and simvastatin (1.02%). Of the 100 readmitted patients, a small percentage (4.06%) suffered another ACS episode in 2017 and 2018.

## Discussion

Demographics and characteristics

Our study findings suggest that ACS was more prevalent in males. This result is consistent with a local prospective registry study that showed that the majority of their samples were males [24]. Another study in Saudi Arabia's southern region found that the frequency of CAD in males was higher than in females [10]. This suggests that males are at a higher risk of suffering from ACS due to the high prevalence of dyslipidemia, smoking, hypertension, and DM [25]. Further, STEMI in our study occurred more frequently in males, and the results are consistent with a study done in Sri Lanka, where the highest proportion of patients with STEMI was males [26]. Moreover, NSTEMI and UA were more prevalent in females, similar to a local study conducted in the southwest region [27]. This can be explained by sex differences in thrombotic and fibrinolytic activities [28].

The usual age groups at presentation in this study were between 55 and 64 years, followed by 45 and 54 years. These findings are in agreement with a prior study conducted in Saudi Arabia's northern region, which showed that most of the cases of ACS were found among people in the 56-65 years and 46-55 years age group [29]. In contrast, the EuroHeart ACS survey conducted in a broad region in Europe and the Mediterranean Basin showed that most patients were between 65 and 74 years [30]. However, this survey did not include Saudi Arabia, and the average age of our study participants at the time of presentation was a decade younger than that reported globally. This might be due to various reasons, including a lack of evaluation by healthcare workers and inadequate understanding of primary care physicians regarding treatment, advanced therapies, and new technologies to aid in managing cardiovascular risk factors [31]. In addition, an increase in urbanization in Saudi Arabia may have profound implications for healthcare services and resource utilization, as well as the accessibility of healthcare facilities [31]. All aforementioned factors may contribute to poor risk factor management and the onset of ACS at a younger age.

Medical history and coronary risk factors

Concerning patients’ medical history, DM, dyslipidemia, and hypertension were the most frequent CAD risk factors. This is in agreement with previous local studies that showed that the majority of patients with ACS had DM, hypertension, and dyslipidemia [32]. In contrast, a study conducted in India discussing ACS-related risk factors within a population reported that DM and hypertension affected a smaller proportion of patients compared to our study [33]. Furthermore, obesity was a significantly common risk factor in our study, accounting for approximately one-quarter of cases, compared with a minority of cases observed in a study conducted in Europe addressing the impact of age on obesity in relation to ACS [34]. This variation suggests that a sedentary lifestyle, adopting a Western-pattern diet, and less regular exercise or physical activity have increased the prevalence of obesity in Saudi Arabia [35]. This established a link with the development of DM, dyslipidemia, and hypertension [36].

In this study, the occurrence of previous ACS episodes was higher among female patients; previous studies showed a lower incidence in female patients [37]. The high mean age of female patients in both studies is a reasonable explanation (64 and 73 years) [37]. Moreover, this could be attributed to the hormonal changes in menopause, which is a risk factor for ACS in females [38].

Lipid parameters at admission

Regarding the lipid profile, we found that the mean of lipid parameters at admission varied depending on sex. Males had significantly higher LDL-C, TC, and TG levels, whereas HDL-C values were higher in female patients. Our findings are consistent with those of prior studies [39-41]. In contrast, previous studies in a Polish population showed that high LDL-C levels were significantly higher in females compared to males (P = 0.033); additionally, other lipid profile components were found to be less controlled in females than in males [42]. Moreover, according to Esteghamati et al., female patients had greater mean TG levels than male patients [43]. These disparities support the theory that genetic differences could explain variances in sex inequality, body fat distribution, lifestyle, and nutritional habits among the nation-states where the research was conducted [39].

Lipid parameters at follow-up

Although lipid-lowering statins were administered to most of the participants in this study at the time of discharge, only 161 of 615 participants returned to the hospital for a follow-up visit. At follow-up, the mean LDL-C, TC, and TG levels were significantly lower than those at admission. Our findings are consistent with those of numerous clinical trials that have demonstrated the benefit of statin therapy with respect to cardiovascular events among patients with ACS [44]. In patients with ACS, dyslipidemia is common and is considered a treatment focus, with clinical trials and meta-analyses increasingly supporting early, intense, and ongoing statin treatment in patients with ACS, as it reduces coronary plaque burden and lowers the risk of cardiovascular mortality and morbidity [45].

The therapeutic target of LDL-C < 1.8 mmol/L (<70 mg/dl) was achieved in only 45 of 161 patients in our study. This is consistent with prior research findings in patients with ACS, which revealed that the goal of LDL-C ≤ 1.8 mmol/L (≤70 mg/dl) was achieved in less than half of the patients [46]. Furthermore, according to another study, only 44 of 242 patients with ACS achieved an LDL-C < 1.8 mmol/L (<70 mg/dL) [47]. Several studies indicated that lowering LDL-C to <1.8 mmol/L (<70 mg/dl) as part of secondary prevention improves the prognosis of individuals with ACS [48]. Lowering LDL-C decreases cardiovascular morbidity and mortality in individuals with atherosclerotic CVD, with a therapeutic effect corresponding to the extent of LDL-C reduction [49,50]. However, these recommendations are only partially applied in daily practice. This can be attributed to several factors, including inadequate treatment intensity, poor patient compliance, and adverse effects associated with high statin dosages [51].

Lipid-lowering therapy

In our study, most patients were prescribed atorvastatin (20 or 40 mg/day) or rosuvastatin (20 or 40 mg/day) during hospitalization. This is in line with the literature as atorvastatin and rosuvastatin are the two most commonly recommended medications for hypercholesterolemia and are regularly used to treat individuals with ACS [52]. Cholesterol guidelines of the American College of Cardiology and American Heart Association have designated atorvastatin doses of ≥40 mg/day or rosuvastatin doses of ≥20 mg/day as high-intensity statins [53]. A previous meta-analysis of individual participant data from randomized trials reported that compared with less intensive regimens, high-intensity statins significantly decreased (15% reduction) major vascular events, with substantial reductions in the incidence of coronary mortality or non-fatal myocardial infarction of 13% [54]. This is in line with expert recommendations, which recommend initiating intense lipid-lowering therapy during the first one to four days following ACS [55].

Furthermore, earlier studies reported that doubling the dose of a statin (atorvastatin 40 mg/day to 80 mg/day) will facilitate a further (6%) reduction in LDL-C, and given the well-established link between lowered LDL-C and outcomes, an 80 mg/day dose will further improve outcomes [56-59]. Additionally, the results of previously completed trials supported the favorable safety profile of atorvastatin at the highest dose [60]. Moreover, the medical team generally determines the proper statin dose in the hospital. In specific situations, such as cases of previous intolerance or abnormal liver function tests, secondary or primary care teams may up-titrate the dose as tolerated, from an initial low dose after discharge [59].

Procedures performed

We found that procedures performed on patients in CCU and those with cardiac diseases included the use of an ECG in most cases. An ECG is used to display the heart’s electrical activity to establish the diagnosis [61]. In addition, cardiac biomarkers are essential indicators of heart damage. In ACS, an increase in these cardiac enzymes provides further diagnostic relevance [62]. Previous studies have suggested that ECGs proved invaluable in the CCU setting [63], as was the case in the CCU of KAUH.

Our findings imply that invasive procedures, such as PCI, are commonly used in the CCU. A study published in the *International Journal of Cardiology* concluded that PCI was the most appropriate measure for patients with STEMI, as the majority of patients over 60 years of age underwent the procedure [64]. PCI was used as both a diagnostic and therapeutic tool, further justifying the critical nature of this intervention in cases of myocardial infarction [65,66]. It is important to note that PCI is also used as a treatment option for patients with ACS because placing a stent in the coronary arteries may lower the risk of mortality by approximately 30% [67].

Echocardiography findings

Echocardiography was performed in almost all patients in our study for the determination of LVEF [68]. Echocardiography is considered reliable for establishing ACS prognosis [69]. The patients in our study demonstrated varying degrees of LV function, as many of them had different risk factors influencing their ejection fraction, and our figures were comparable to those of other studies [69].

Regarding the type of ACS, patients with STEMI, in terms of disease severity, had varying levels of LVEF. However, the patients with NSTEMI in our study were classified as having the worst result, with an ejection fraction < 30%. Moreover, a 2020 study published in the *European Journal of Preventive Cardiology* found that the number of patients with STEMI with a <45% ejection fraction was significantly higher than patients with NSTEMI in both sexes [70]. These data suggest the presence of unique environmental and genetic risk factors across patients in different countries [71,72]. Our patients have displayed intriguing outcomes that may not have been considered possible before conducting our study.

These discrepancies might seem unrealistic at first glance; however, with further understanding of the different risk factors in patients with ACS, it becomes clear that patients in the CCU exhibit mixed results that are open for interpretation. Overall, the importance of invasive and non-invasive procedures in the CCU of KAUH cannot be underestimated, as they prove to be vital tools in guiding physicians toward the correct diagnosis and treatment of cardiac patients.

Hospital outcomes

Our study indicates a high recovery rate from ACS. Of the patients in our study, 99.5% of patients recovered and were discharged compared to 97.2% of patients who recovered and were discharged from a secondary care center in the southern region of Saudi Arabia during the same period as our study [10]. In another study in Kerala, India, the recovery rate without hospital adverse events was 94.3% [73]. Based on a previous study conducted at Tehran Medical Center, the mortality rate was significantly higher in a low socio-economic status (SES) group than in a high SES group [74]; this emphasizes that SES may play a major role in these disparities.

Our results showed that reinfarction was the most common adverse event in the patients. The proportion of patients readmitted to the CCU was mainly patients with diabetes and hypertension. A previous study conducted in a medical center in Sweden showed that the most common adverse event among patients with diabetes was reinfarction and concluded that the infarction occurrence rate is doubled in diabetic patients compared to non-diabetic patients [75]. Similar observations have been made in previous studies [76-78]. According to a previous study, patients with diabetes were more likely to develop ischemic cardiomyopathy than those without diabetes [78]. Hypertension is common in patients with diabetes, and a previous study in Switzerland revealed that in a one-year follow-up of patients with ACS, patients with hypertension had worse unmodified outcomes with 65% more chances of developing reinfarction than patients without hypertension [79]. It has been shown that in patients with diabetes and hypertension, the prevalence of macro-vascular consequences (myocardial infarction and stroke) is much higher than in those without hypertension [80].

Our research data show that the percentage of readmission among females was higher than that among males, a finding similar to that obtained in a study conducted in multiple centers worldwide that found a higher percentage of readmission in females than males [81]. A previous study showed that cardiovascular medications might have different effects on men and women because of variations in body composition, fluctuations in endogenous sex hormone levels (female monthly cycle and gestation), the pharmacokinetics/pharmacodynamics characteristics of some medicines, or the use of hormone replacement treatment or oral contraceptives [41,82].

Limitations

There are certain limitations to our study. This research was a retrospective analysis with concerns regarding the completeness and accuracy of the data recorded. In addition, this was a single-center study; hence, there are potential differences compared with other regions or countries. The follow-up period in this study was not equivalent for all patients; more precise timing is required to further support the findings regarding lipid parameters at follow-up obtained from this study. We conducted this study among inpatients of a tertiary teaching center. As the quality of management and clinical outcomes are not ubiquitous throughout Saudi Arabia, our results could only reflect a higher level of medical care than other studies conducted in the same region.

## Conclusions

In this study, we included patients with ACS admitted to the CCU at KAUH in 2017 and aimed to analyze patient demographics, risk factors, investigations, and outcomes. Our results revealed that a significant proportion of patients were a decade younger than what has been recorded worldwide. The most prevalent final diagnosis was a STEMI. Moreover, hypertension, DM, and dyslipidemia were the most frequent modifiable cardiovascular risk factors. Reinfarction was the most common adverse event, with a strong association with hypertension and DM.

Nevertheless, the recovery rate in this study was higher than that in other countries. At follow-up, many patients did not meet the target lipid levels. The younger presentation age of our population needs critical consideration and more strict preventive interventions, such as lifestyle modifications and evidence-based treatments for CVD risk factors to decrease ACS events, thereby, decreasing the burden on the CCUs as well as CVD morbidity and mortality. However, the rate of ACS admission was much lower during the COVID-19 pandemic period, with a significant link with COVID-19 prevalence. As a result, we looked at patients admitted during 2017 to avert the pandemic's impact on healthcare systems worldwide. In addition, Saudi Arabia is a country with rapidly evolving cardiovascular demographics; therefore, more research is necessary to understand the presentations, risk factors, management settings, and clinical outcomes of ACS at the regional level since they may differ across regions and with time.
